# Stevioside methanol tetra­solvate

**DOI:** 10.1107/S1600536813003954

**Published:** 2013-02-20

**Authors:** Yunshan Wu, Douglas L. Rodenburg, Mohamed A. Ibrahim, James D. McChesney, Mitchell A. Avery

**Affiliations:** a Department of Medicinal Chemistry, University of Mississippi, 417 Faser Hall, University, MS 38677, USA; b Ironstone Separations, Inc., 147 County Road 245, Etta, Mississippi 38627, USA; c Department of Chemistry and Biochemistry, University of Mississippi, 417 Faser Hall, University, MS 38677, USA

## Abstract

Stevioside is a naturally occurring diterpenoid glycoside in *Stevia rebaudiana* Bertoni. The title compound, C_38_H_60_O_18_·4CH_3_OH, crystallized as its methanol tetrasolvate. Stevioside consists of an aglycone steviol (a tetra­cyclic diterpene in which the four-fused-ring system consists of three six-membered rings and one five-membered ring) and a sugar part (three glucose units). A weak intra­molecular O—H⋯O hydrogen bond occurs. In the crystal, the methanol mol­ecules participate in a two-dimensional hydrogen-bonded network parallel to *b* axis with the sugars and together they form a hydrophilic tunnel which encloses the lipophilic part of the molecule.

## Related literature

For low-calorie sweeteners, see: Bertoni (1905 ▶); Kinghorn (2002 ▶). For the Joint FAO/WHO Expert Committee on Food Additives, see: JECFA (2010 ▶). For the US Food and Drug Administration granted regulatory acceptance of Rebaudioside A, see: FDA (2008 ▶) and of mixtures of steviol glycosides, see: FDA (2010 ▶). For European Union approved steviol glycosides, see: OJ L (2011 ▶). For commercilization of glycoside sweeteners from S. rebaudiana, see: Prakash *et al.* (2008 ▶); Wölwer-Rieck (2012 ▶). For a related structure, see: Prakash & Upreti (2011 ▶). For puckering parameters, see: Cremer & Pople (1975 ▶).
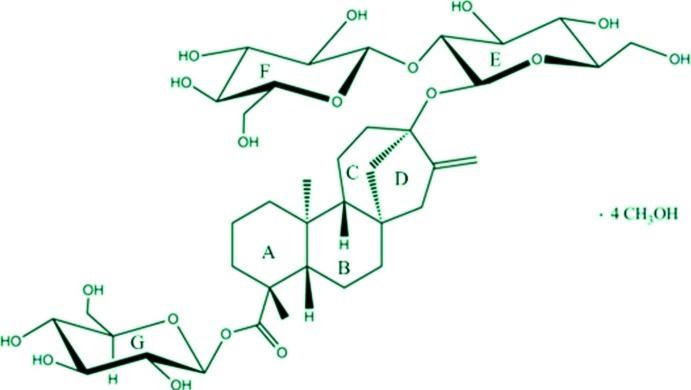


## Experimental

### 

#### Crystal data

C_38_H_60_O_18_·4CH_4_O
*M* *_r_* = 933.03
Monoclinic, 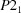

*a* = 15.0413 (2) Å
*b* = 7.7866 (1) Å
*c* = 19.6443 (3) Å
β = 96.231 (1)°
*V* = 2287.16 (5) Å^3^
*Z* = 2
Cu *K*α radiation
μ = 0.92 mm^−1^
*T* = 100 K
0.42 × 0.14 × 0.11 mm

#### Data collection

Bruker APEXII CCD diffractometer
Absorption correction: multi-scan (*SADABS*; Bruker, 1999 ▶) *T*_min_ = 0.699, *T*_max_ = 0.906
29032 measured reflections
7325 independent reflections
7174 reflections with *I* > 2σ(*I*)
*R*_int_ = 0.031

#### Refinement

*R*[*F* ^2^ > 2σ(*F* ^2^)] = 0.030
*wR*(*F* ^2^) = 0.076
*S* = 1.05
7325 reflections
612 parameters
1 restraint
H-atom parameters constrained
Δρ_max_ = 0.34 e Å^−3^
Δρ_min_ = −0.35 e Å^−3^
Absolute structure: Flack (1983 ▶), 2830 Friedel pairs
Flack parameter: 0.11 (9)

### 

Data collection: *APEX2* (Bruker, 2009 ▶); cell refinement: *SAINT* (Bruker, 2009 ▶); data reduction: *SAINT*; program(s) used to solve structure: *SHELXS97* (Sheldrick, 2008 ▶); program(s) used to refine structure: *SHELXL97* (Sheldrick, 2008 ▶); molecular graphics: *ORTEP-3 for Windows* (Farrugia, 2012 ▶); software used to prepare material for publication: *SHELXTL* (Sheldrick, 2008 ▶).

## Supplementary Material

Crystal structure: contains datablock(s) I, global. DOI: 10.1107/S1600536813003954/jj2161sup1.cif

Structure factors: contains datablock(s) I. DOI: 10.1107/S1600536813003954/jj2161Isup2.hkl

Additional supplementary materials:  crystallographic information; 3D view; checkCIF report

## Figures and Tables

**Table 1:** Hydrogen-bond geometry (Å, °)

*D*—H⋯*A*	*D*—H	H⋯*A*	*D*⋯*A*	*D*—H⋯*A*
O4*S*—H4*OS*⋯O13^i^	0.84	1.90	2.7330 (17)	169
O3*S*—H3*OS*⋯O11^i^	0.84	1.88	2.7115 (19)	170
O2*S*—H2*OS*⋯O8^ii^	0.84	1.90	2.7381 (19)	177
O18—H18*O*⋯O2*S* ^iii^	0.84	1.86	2.6857 (19)	167
O15—H15*O*⋯O10^iv^	0.84	2.22	3.0000 (19)	154
O14—H14*O*⋯O18^v^	0.84	1.91	2.6983 (19)	157
O13—H13*O*⋯O7^iii^	0.84	1.91	2.7080 (19)	158
O11—H11*O*⋯O3*S* ^vi^	0.84	1.89	2.7248 (17)	169
O10—H10*O*⋯O16^iv^	0.84	1.88	2.7217 (18)	177
O7—H7*O*⋯O1*S* ^v^	0.84	1.96	2.777 (2)	166
O1*S*—H1*OS*⋯O1	0.84	2.08	2.860 (2)	154
O16—H16*O*⋯O4*S*	0.84	1.83	2.6558 (17)	168
O5—H5*O*⋯O3*S*	0.84	1.99	2.8081 (17)	166
O4—H4*O*⋯O15	0.86	2.38	3.2012 (17)	160
O3—H3*O*⋯O15	0.84	1.94	2.7569 (17)	165

Symmetry codes: (i) 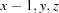; (ii) 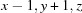; (iii) 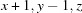; (iv) 
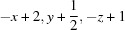
; (v) 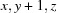; (vi) 
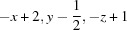
.

**Fig. 1.**
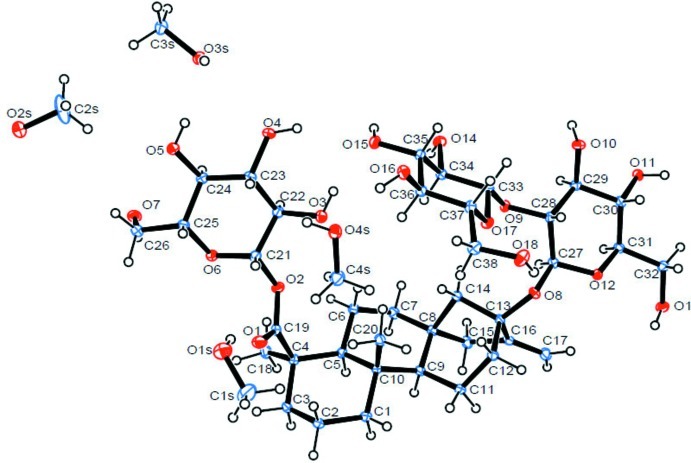
Molecular structure of stevioside methanol solvate with displacement ellipsoids at 30% probability level.

**Fig. 2.**
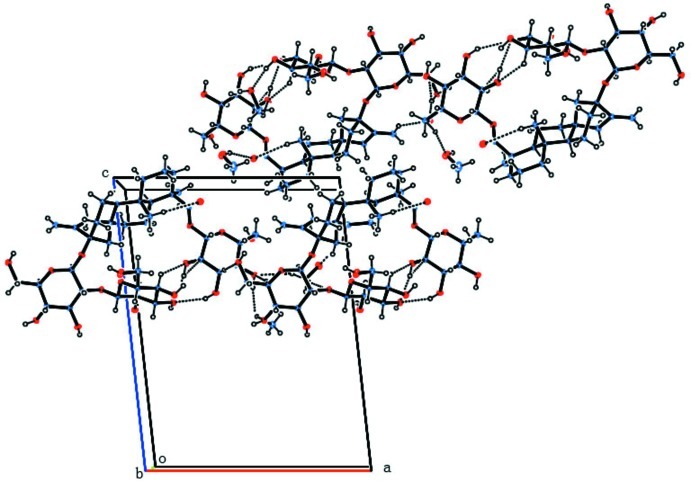
Packing diagram of the title compound viewed along the b axis. Dashed lines indicate O—H···O hydrogen bonds and weak O—H···O intermolecular interactions. Hydrogen atoms not involved in hydrogen bonding have been deleted for clarity.

## supplementary crystallographic information

### Comment

Stevia rebaudiana Bertoni (Asteraceae), first identified in 1905 by the botanist Bertoni, is a perennial bush indigenous to the mountainous regions of Paraguay and Brazil, and is well known to produce a mixture of high-potency sweet compounds that have been in the market since the 1970's as low-calorie sweeteners (Bertoni, 1905; Kinghorn, 2002). Stevioside is one of these low-calorie sweeteners. The native cultures of Paraguay and Brazil have safely used stevia for sweetening for centuries. The Joint FAO/WHO Expert Committee on Food Additives has established a monograph for Steviol Glycosides (JECFA, 2010). The United States Food and Drug Administration granted regulatory acceptance of Rebaudioside A (FDA, 2008) and mixtures of steviol glycosides (FDA, 2010) followed by the European Union approved steviol glycosides for marketing (OJ, 2011). The world demand for Stevia leaves is expected to exceed 6–8 million metric tonnes in the next 10 years. Stevioside and Rebaudioside A, also knownas Rebiana, are the major glycoside sweeteners from S. rebaudiana which have been commercialized for human consumption throughout the world (Prakash *et al.* 2008; Wölwer-Rieck, 2012).

Stevioside has several different polymorph forms (Prakash & Upreti, 2011). These polymorph forms include methanol or ethanol solvate, hydrate and amorphous. These different polymorphic forms would affect the physical properties like melting point, solubility and density. The powder X-ray patterns of these forms have been documented; however, no single-crystal results have been reported so far. Here we report the structure of stevioside methanol solvate.

The asymmetric unit contains one stevioside and four methanol molecules (Fig. 1). Stevioside has an aglycone portion and a sugar portion. The aglycone is a tetracyclic diterpene in which the four fused ring system consists of three six membered rings and one five membered ring. Rings A, B and C form chair conformations with the puckering parameter (Cremer & Pople, 1975) Q = 0.550 (2)Å, θ = 175.6 (2)° and φ= 255 (3)°; Q = 0.5753 (17)Å, θ = 166.72 (17)° and φ= 191.3 (8)°; Q = 0.6355 (17)Å, θ = 24.09 (16)° and φ= 281.3 (4)°, respectively. The five-membered ring D forms an envelope conformation with the puckering parameter Q = 0.4791 (19)Å, φ= 24.6 (2)°. These ring systems form the hydrophobic part of the molecule. The three sugars are all β-D glucopyranoses (E, F, G) and they all form chair conformations with the puckering parameters Q = 0.5660 (17)Å, θ = 9.94 (16)°, φ = 4.3 (10)°, Q = 0.6026 (17)Å, θ = 3.01 (16)°, φ= 72 (3)° and Q = 0.5861 (19) Å, θ =1.56 (18)°, φ= 358 (7)°, respectively. One sugar is attached to the carboxylic acid functional group at C4 while the other two sugars are linked as 2ρightarrow1 and attached to C13. The sugar attached to the carboxylic group at C4 has been shown to be linked on an axial position instead of an equatorial position.

Stevioside and four methanol molecules were stablized by an extensive hydrogen bonding network and a weak O4—H4O···O15 intramolecular interaction (Table 1). These hydrogen bonds and weak interaction further link two adjacent asymmetric units to form a hydrophilic layer. The aglycon part of the stevioside forms a hydrophobic nucleus. This bilayer system acts like an oil in water system (Fig. 2). This is believed to increase the solubility of stevioside.

### Experimental

The title compound was isolated by selective crystallization from a commercially available extract of Stevia rebaudiana. The composition of the extract was approximately 42% Rebaudioside A, 31% Stevioside, 10% Rebaudioside C by HPLC analysis with the remaining 13% of the extract being minor steviol glycoside components. Stevioside was crystallized from a 90/10 *v*/*v* methanol/water mixture containing dissolved extract. The isolated Stevioside was then re-crystallized from methanol to greater than 95% purity, and further re-crystallized to produce crystals suitable for crystallography.

### Refinement

All the H atoms were placed in their calculated positions and then refined using the riding model with Atom—H lengths of 1.00Å (CH), 0.99Å (CH_2_) or 0.98Å (CH_3_). Isotropic displacement parameters for these atoms were set to 1.2 (CH, CH_2_) or 1.5 (CH_3_) times *U*_eq_ of the parent atom. H atoms on hydroxyl were initially found in the difference map and then constrained to their parent atoms as riding model with *U*_iso_(H) = 1.2Ueq of parent atoms.

### Crystal data

**Table:**

C_38_H_60_O_18_·4CH_4_O	*F*(000) = 1008
*M**_r_* = 933.03	*D*_x_ = 1.355 Mg m^−^^3^
Monoclinic, *P*2_1_	Cu *K*α radiation, λ = 1.54178 Å
Hall symbol: P 2yb	Cell parameters from 9008 reflections
*a* = 15.0413 (2) Å	θ = 2.3–68.8°
*b* = 7.7866 (1) Å	µ = 0.92 mm^−^^1^
*c* = 19.6443 (3) Å	*T* = 100 K
β = 96.231 (1)°	Block, colourless
*V* = 2287.16 (5) Å^3^	0.42 × 0.14 × 0.11 mm
*Z* = 2	

### Data collection

**Table:**

Bruker APEXII CCD diffractometer	7325 independent reflections
Radiation source: fine-focus sealed tube	7174 reflections with *I* > 2σ(*I*)
Graphite monochromator	*R*_int_ = 0.031
φ and ω scans	θ_max_ = 69.4°, θ_min_ = 2.3°
Absorption correction: multi-scan (*SADABS*; Bruker, 1999)	*h* = −17→18
*T*_min_ = 0.699, *T*_max_ = 0.906	*k* = −9→9
29032 measured reflections	*l* = −23→22

### Refinement

**Table:**

Refinement on *F*^2^	Secondary atom site location: difference Fourier map
Least-squares matrix: full	Hydrogen site location: inferred from neighbouring sites
*R*[*F*^2^ > 2σ(*F*^2^)] = 0.030	H-atom parameters constrained
*wR*(*F*^2^) = 0.076	*w* = 1/[σ^2^(*F*_o_^2^) + (0.0397*P*)^2^ + 0.7153*P*] where *P* = (*F*_o_^2^ + 2*F*_c_^2^)/3
*S* = 1.05	(Δ/σ)_max_ = 0.007
7325 reflections	Δρ_max_ = 0.34 e Å^−^^3^
612 parameters	Δρ_min_ = −0.35 e Å^−^^3^
1 restraint	Absolute structure: Flack (1983), 2830 Friedel pairs
Primary atom site location: structure-invariant direct methods	Flack parameter: 0.11 (9)

### Special details

**Table:**

Geometry. All e.s.d.'s (except the e.s.d. in the dihedral angle between two l.s. planes) are estimated using the full covariance matrix. The cell e.s.d.'s are taken into account individually in the estimation of e.s.d.'s in distances, angles and torsion angles; correlations between e.s.d.'s in cell parameters are only used when they are defined by crystal symmetry. An approximate (isotropic) treatment of cell e.s.d.'s is used for estimating e.s.d.'s involving l.s. planes.
Refinement. Refinement of *F*^2^ against ALL reflections. The weighted *R*-factor *wR* and goodness of fit *S* are based on *F*^2^, conventional *R*-factors *R* are based on *F*, with *F* set to zero for negative *F*^2^. The threshold expression of *F*^2^ > σ(*F*^2^) is used only for calculating *R*-factors(gt) *etc*. and is not relevant to the choice of reflections for refinement. *R*-factors based on *F*^2^ are statistically about twice as large as those based on *F*, and *R*- factors based on ALL data will be even larger.

### Fractional atomic coordinates and isotropic or equivalent isotropic displacement parameters (Å^2^)

**Table:**

	*x*	*y*	*z*	*U*_iso_*/*U*_eq_	
O1	0.62491 (8)	0.4165 (2)	0.08527 (6)	0.0244 (3)	
O2	0.68765 (8)	0.61200 (18)	0.15946 (6)	0.0195 (3)	
O3	0.71977 (9)	0.48607 (19)	0.29267 (6)	0.0244 (3)	
H3O	0.7351	0.4873	0.3351	0.027 (6)*	
O4	0.60558 (8)	0.63638 (18)	0.39001 (6)	0.0210 (3)	
H4O	0.6531	0.5898	0.4105	0.025*	
O5	0.42495 (8)	0.70491 (19)	0.32725 (6)	0.0211 (3)	
H5O	0.4398	0.7048	0.3697	0.038 (7)*	
O6	0.55057 (8)	0.70459 (18)	0.17930 (6)	0.0186 (3)	
O7	0.42457 (9)	0.98586 (18)	0.19277 (6)	0.0240 (3)	
H7O	0.4483	1.0207	0.1585	0.044 (8)*	
O8	1.15787 (7)	0.19208 (16)	0.27168 (6)	0.0155 (2)	
O9	1.09935 (7)	0.37255 (16)	0.38503 (6)	0.0148 (2)	
O10	1.21599 (8)	0.29684 (17)	0.51152 (6)	0.0182 (3)	
H10O	1.2178	0.3842	0.5367	0.029 (6)*	
O11	1.40099 (7)	0.36487 (17)	0.48897 (6)	0.0173 (3)	
H11O	1.4452	0.3034	0.5027	0.031 (6)*	
O12	1.29790 (7)	0.19179 (17)	0.32430 (6)	0.0153 (2)	
O13	1.48163 (8)	0.19920 (19)	0.29735 (6)	0.0214 (3)	
H13O	1.4507	0.1371	0.2689	0.030 (6)*	
O14	0.97163 (8)	0.56441 (18)	0.44911 (6)	0.0199 (3)	
H14O	0.9822	0.6494	0.4249	0.027 (6)*	
O15	0.79336 (8)	0.44608 (17)	0.42648 (6)	0.0192 (3)	
H15O	0.8094	0.5406	0.4444	0.037 (7)*	
O16	0.77180 (8)	0.08335 (18)	0.40865 (6)	0.0193 (3)	
H16O	0.7280	0.0822	0.3782	0.036 (7)*	
O17	0.99938 (7)	0.15411 (17)	0.36344 (6)	0.0168 (2)	
O18	0.97793 (9)	−0.21387 (18)	0.34418 (7)	0.0245 (3)	
H18O	1.0228	−0.1788	0.3262	0.034 (7)*	
C1	0.86165 (11)	0.2886 (3)	0.01423 (9)	0.0217 (4)	
H1A	0.8969	0.3422	−0.0198	0.026*	
H1B	0.8785	0.1657	0.0178	0.026*	
C2	0.76225 (12)	0.3018 (3)	−0.01169 (9)	0.0227 (4)	
H2A	0.7513	0.2481	−0.0575	0.027*	
H2B	0.7266	0.2390	0.0198	0.027*	
C3	0.73284 (12)	0.4887 (3)	−0.01607 (9)	0.0216 (4)	
H3A	0.6682	0.4935	−0.0321	0.026*	
H3B	0.7653	0.5482	−0.0504	0.026*	
C4	0.74981 (11)	0.5848 (3)	0.05294 (8)	0.0182 (4)	
C5	0.85041 (11)	0.5628 (2)	0.08160 (8)	0.0160 (3)	
H5A	0.8837	0.6196	0.0464	0.019*	
C6	0.88173 (11)	0.6594 (2)	0.14795 (9)	0.0170 (3)	
H6A	0.8536	0.7744	0.1472	0.020*	
H6B	0.8637	0.5954	0.1878	0.020*	
C7	0.98361 (11)	0.6776 (2)	0.15429 (9)	0.0181 (4)	
H7A	1.0035	0.7379	0.1976	0.022*	
H7B	1.0000	0.7501	0.1162	0.022*	
C8	1.03385 (11)	0.5064 (2)	0.15317 (8)	0.0162 (4)	
C9	0.99186 (11)	0.3896 (2)	0.09413 (8)	0.0162 (4)	
H9	1.0066	0.4488	0.0516	0.019*	
C10	0.88693 (11)	0.3753 (2)	0.08459 (8)	0.0159 (4)	
C11	1.04220 (11)	0.2157 (3)	0.09361 (9)	0.0190 (4)	
H11A	1.0016	0.1303	0.0692	0.023*	
H11B	1.0938	0.2305	0.0669	0.023*	
C12	1.07688 (11)	0.1410 (2)	0.16431 (8)	0.0172 (4)	
H12A	1.0267	0.0880	0.1854	0.021*	
H12B	1.1217	0.0506	0.1586	0.021*	
C13	1.11932 (10)	0.2821 (2)	0.21134 (8)	0.0150 (3)	
C14	1.04629 (11)	0.4127 (2)	0.22327 (8)	0.0157 (3)	
H14A	1.0663	0.4927	0.2610	0.019*	
H14B	0.9905	0.3551	0.2333	0.019*	
C15	1.13387 (11)	0.5393 (3)	0.14351 (9)	0.0195 (4)	
H15A	1.1415	0.5508	0.0943	0.023*	
H15B	1.1554	0.6458	0.1674	0.023*	
C16	1.18488 (11)	0.3854 (2)	0.17396 (8)	0.0178 (4)	
C17	1.26902 (12)	0.3420 (3)	0.16766 (9)	0.0226 (4)	
H17A	1.3043	0.4117	0.1414	0.027*	
H17B	1.2939	0.2413	0.1894	0.027*	
C18	0.72959 (12)	0.7763 (3)	0.03850 (9)	0.0235 (4)	
H18A	0.6697	0.7880	0.0139	0.035*	
H18B	0.7739	0.8235	0.0105	0.035*	
H18C	0.7324	0.8391	0.0819	0.035*	
C19	0.68125 (11)	0.5238 (2)	0.09989 (9)	0.0178 (4)	
C20	0.84897 (11)	0.2649 (3)	0.13979 (9)	0.0185 (4)	
H20A	0.7851	0.2448	0.1268	0.028*	
H20B	0.8572	0.3252	0.1838	0.028*	
H20C	0.8805	0.1546	0.1439	0.028*	
C21	0.61780 (11)	0.5849 (3)	0.20199 (8)	0.0178 (4)	
H21	0.5942	0.4651	0.1962	0.021*	
C22	0.65521 (11)	0.6156 (2)	0.27567 (9)	0.0180 (4)	
H22	0.6838	0.7316	0.2805	0.022*	
C23	0.57712 (11)	0.6046 (2)	0.31942 (8)	0.0173 (4)	
H23	0.5529	0.4850	0.3155	0.021*	
C24	0.50219 (11)	0.7268 (2)	0.29257 (9)	0.0168 (4)	
H24	0.5237	0.8481	0.2979	0.020*	
C25	0.47384 (11)	0.6895 (3)	0.21689 (8)	0.0179 (4)	
H25	0.4510	0.5689	0.2125	0.021*	
C26	0.40226 (12)	0.8090 (3)	0.18420 (9)	0.0219 (4)	
H26A	0.3917	0.7832	0.1346	0.026*	
H26B	0.3458	0.7867	0.2044	0.026*	
C27	1.21543 (10)	0.2811 (2)	0.32032 (8)	0.0149 (3)	
H27	1.2228	0.4031	0.3061	0.018*	
C28	1.17908 (10)	0.2715 (2)	0.39009 (8)	0.0139 (3)	
H28	1.1642	0.1496	0.4002	0.017*	
C29	1.24630 (11)	0.3407 (2)	0.44738 (8)	0.0147 (3)	
H29	1.2490	0.4686	0.4435	0.018*	
C30	1.33947 (11)	0.2672 (2)	0.44328 (8)	0.0155 (3)	
H30	1.3406	0.1448	0.4588	0.019*	
C31	1.36540 (10)	0.2752 (3)	0.37006 (8)	0.0159 (3)	
H31	1.3717	0.3976	0.3560	0.019*	
C32	1.45146 (11)	0.1791 (3)	0.36342 (9)	0.0194 (4)	
H32A	1.4984	0.2212	0.3987	0.023*	
H32B	1.4421	0.0554	0.3720	0.023*	
C33	1.02608 (11)	0.2918 (2)	0.40889 (8)	0.0151 (3)	
H33	1.0417	0.2483	0.4566	0.018*	
C34	0.94986 (11)	0.4208 (2)	0.40639 (8)	0.0161 (3)	
H34	0.9351	0.4611	0.3582	0.019*	
C35	0.86824 (11)	0.3324 (2)	0.43006 (8)	0.0163 (3)	
H35	0.8828	0.2929	0.4784	0.020*	
C36	0.84391 (10)	0.1772 (2)	0.38452 (8)	0.0156 (3)	
H36	0.8255	0.2173	0.3367	0.019*	
C37	0.92545 (11)	0.0596 (2)	0.38472 (9)	0.0169 (4)	
H37	0.9419	0.0140	0.4320	0.020*	
C38	0.90912 (12)	−0.0890 (3)	0.33518 (9)	0.0210 (4)	
H38A	0.8513	−0.1438	0.3418	0.025*	
H38B	0.9049	−0.0447	0.2877	0.025*	
O1S	0.49100 (10)	0.1557 (2)	0.08470 (7)	0.0325 (3)	
H1OS	0.5215	0.2438	0.0955	0.058 (9)*	
C1S	0.53475 (15)	0.0556 (4)	0.03935 (13)	0.0416 (6)	
H1S1	0.5290	0.1102	−0.0059	0.062*	
H1S2	0.5077	−0.0590	0.0357	0.062*	
H1S3	0.5982	0.0455	0.0565	0.062*	
O2S	0.13208 (8)	0.84829 (18)	0.29127 (7)	0.0236 (3)	
H2OS	0.1415	0.9527	0.2840	0.042 (8)*	
C2S	0.20002 (17)	0.7843 (3)	0.34007 (16)	0.0520 (7)	
H2S1	0.2571	0.7839	0.3204	0.078*	
H2S2	0.1851	0.6670	0.3528	0.078*	
H2S3	0.2049	0.8577	0.3808	0.078*	
O3S	0.44234 (8)	0.69960 (18)	0.47101 (6)	0.0201 (3)	
H3OS	0.4326	0.5935	0.4724	0.025 (6)*	
C3S	0.37019 (12)	0.7913 (3)	0.49773 (10)	0.0241 (4)	
H3S1	0.3397	0.7148	0.5273	0.036*	
H3S2	0.3276	0.8308	0.4597	0.036*	
H3S3	0.3944	0.8905	0.5243	0.036*	
O4S	0.65006 (8)	0.0658 (2)	0.30034 (6)	0.0234 (3)	
H4OS	0.6015	0.1184	0.3014	0.031 (6)*	
C4S	0.67878 (13)	0.0804 (3)	0.23360 (10)	0.0266 (4)	
H4S1	0.6905	0.2012	0.2239	0.040*	
H4S2	0.6319	0.0364	0.1995	0.040*	
H4S3	0.7336	0.0134	0.2316	0.040*	

### Atomic displacement parameters (Å^2^)

**Table:**

	*U*^11^	*U*^22^	*U*^33^	*U*^12^	*U*^13^	*U*^23^
O1	0.0229 (6)	0.0298 (8)	0.0209 (6)	−0.0046 (6)	0.0048 (5)	−0.0063 (6)
O2	0.0170 (6)	0.0247 (7)	0.0173 (6)	−0.0016 (5)	0.0048 (4)	−0.0035 (5)
O3	0.0251 (6)	0.0284 (8)	0.0190 (6)	0.0107 (6)	−0.0008 (5)	−0.0024 (6)
O4	0.0218 (6)	0.0284 (7)	0.0123 (5)	0.0056 (5)	−0.0004 (4)	−0.0015 (5)
O5	0.0172 (6)	0.0294 (7)	0.0170 (6)	0.0008 (6)	0.0036 (4)	0.0005 (6)
O6	0.0174 (6)	0.0236 (7)	0.0152 (5)	0.0021 (5)	0.0030 (4)	0.0007 (5)
O7	0.0308 (7)	0.0232 (7)	0.0179 (6)	0.0057 (6)	0.0017 (5)	0.0013 (5)
O8	0.0165 (5)	0.0155 (6)	0.0138 (5)	−0.0014 (5)	−0.0020 (4)	0.0007 (5)
O9	0.0134 (5)	0.0139 (6)	0.0172 (5)	0.0016 (5)	0.0021 (4)	0.0017 (5)
O10	0.0227 (6)	0.0189 (7)	0.0135 (5)	−0.0007 (5)	0.0047 (5)	−0.0011 (5)
O11	0.0147 (6)	0.0192 (6)	0.0172 (6)	0.0008 (5)	−0.0021 (4)	−0.0032 (5)
O12	0.0123 (5)	0.0173 (6)	0.0160 (5)	0.0009 (5)	0.0000 (4)	−0.0030 (5)
O13	0.0181 (6)	0.0283 (7)	0.0184 (6)	−0.0009 (6)	0.0043 (5)	−0.0048 (6)
O14	0.0222 (6)	0.0186 (7)	0.0190 (6)	0.0002 (5)	0.0023 (5)	−0.0041 (5)
O15	0.0165 (6)	0.0192 (7)	0.0217 (6)	0.0033 (5)	0.0013 (5)	−0.0026 (5)
O16	0.0146 (6)	0.0220 (7)	0.0213 (6)	−0.0027 (5)	0.0024 (5)	0.0053 (5)
O17	0.0153 (5)	0.0176 (6)	0.0181 (5)	−0.0014 (5)	0.0047 (4)	−0.0008 (5)
O18	0.0240 (6)	0.0163 (7)	0.0348 (7)	−0.0001 (6)	0.0106 (5)	0.0011 (6)
C1	0.0192 (8)	0.0303 (11)	0.0151 (8)	0.0038 (8)	−0.0004 (6)	−0.0071 (8)
C2	0.0188 (8)	0.0334 (11)	0.0154 (8)	0.0032 (8)	−0.0011 (6)	−0.0079 (8)
C3	0.0157 (8)	0.0351 (11)	0.0132 (8)	0.0041 (8)	−0.0018 (6)	−0.0022 (8)
C4	0.0155 (8)	0.0231 (10)	0.0160 (8)	0.0036 (7)	0.0013 (6)	−0.0007 (8)
C5	0.0144 (8)	0.0192 (9)	0.0145 (8)	0.0023 (7)	0.0023 (6)	0.0014 (7)
C6	0.0187 (8)	0.0159 (9)	0.0164 (8)	0.0022 (7)	0.0016 (6)	0.0002 (7)
C7	0.0194 (8)	0.0154 (9)	0.0188 (8)	−0.0008 (7)	−0.0005 (6)	0.0014 (7)
C8	0.0172 (8)	0.0178 (9)	0.0136 (8)	−0.0006 (7)	0.0008 (6)	0.0007 (7)
C9	0.0153 (8)	0.0211 (9)	0.0121 (7)	0.0009 (7)	0.0010 (6)	0.0008 (7)
C10	0.0153 (8)	0.0200 (9)	0.0125 (7)	0.0021 (7)	0.0013 (6)	−0.0019 (7)
C11	0.0174 (8)	0.0247 (10)	0.0145 (8)	0.0020 (8)	−0.0003 (6)	−0.0044 (8)
C12	0.0177 (8)	0.0174 (9)	0.0161 (8)	0.0011 (7)	−0.0007 (6)	−0.0028 (7)
C13	0.0148 (7)	0.0161 (9)	0.0136 (7)	0.0005 (7)	−0.0011 (6)	−0.0002 (7)
C14	0.0149 (7)	0.0180 (9)	0.0141 (7)	−0.0005 (7)	0.0013 (6)	0.0002 (7)
C15	0.0178 (8)	0.0213 (10)	0.0191 (8)	−0.0010 (7)	0.0009 (6)	0.0053 (7)
C16	0.0183 (8)	0.0222 (10)	0.0123 (7)	−0.0032 (8)	−0.0010 (6)	−0.0013 (7)
C17	0.0186 (8)	0.0303 (11)	0.0189 (8)	0.0019 (8)	0.0025 (6)	0.0043 (8)
C18	0.0213 (8)	0.0266 (10)	0.0223 (9)	0.0052 (8)	0.0009 (7)	0.0047 (8)
C19	0.0149 (8)	0.0211 (9)	0.0170 (8)	0.0054 (7)	−0.0006 (6)	−0.0002 (7)
C20	0.0158 (8)	0.0199 (9)	0.0193 (8)	−0.0003 (7)	−0.0007 (6)	−0.0004 (7)
C21	0.0162 (8)	0.0194 (9)	0.0184 (8)	0.0015 (7)	0.0039 (6)	−0.0007 (7)
C22	0.0161 (8)	0.0193 (9)	0.0185 (8)	0.0012 (7)	0.0012 (6)	−0.0026 (7)
C23	0.0191 (8)	0.0187 (9)	0.0140 (8)	−0.0010 (7)	0.0017 (6)	−0.0005 (7)
C24	0.0174 (8)	0.0167 (9)	0.0165 (8)	0.0005 (7)	0.0034 (6)	−0.0011 (7)
C25	0.0162 (8)	0.0208 (9)	0.0168 (8)	−0.0019 (8)	0.0026 (6)	−0.0015 (8)
C26	0.0202 (8)	0.0268 (10)	0.0181 (8)	0.0003 (8)	−0.0014 (7)	−0.0004 (8)
C27	0.0146 (7)	0.0138 (8)	0.0159 (8)	−0.0009 (7)	0.0002 (6)	0.0001 (7)
C28	0.0133 (7)	0.0131 (8)	0.0154 (8)	0.0010 (7)	0.0013 (6)	0.0012 (7)
C29	0.0174 (8)	0.0133 (8)	0.0136 (8)	−0.0011 (7)	0.0030 (6)	0.0011 (7)
C30	0.0162 (8)	0.0143 (8)	0.0153 (8)	0.0000 (7)	−0.0013 (6)	0.0010 (7)
C31	0.0147 (8)	0.0178 (9)	0.0147 (8)	−0.0014 (7)	0.0000 (6)	−0.0009 (7)
C32	0.0161 (8)	0.0245 (10)	0.0176 (8)	0.0011 (8)	0.0019 (6)	−0.0026 (8)
C33	0.0158 (7)	0.0173 (9)	0.0124 (7)	−0.0015 (7)	0.0027 (6)	−0.0016 (7)
C34	0.0166 (8)	0.0166 (9)	0.0150 (7)	−0.0005 (7)	0.0008 (6)	0.0006 (7)
C35	0.0145 (7)	0.0197 (9)	0.0148 (7)	0.0030 (7)	0.0024 (6)	0.0013 (7)
C36	0.0139 (7)	0.0179 (9)	0.0153 (7)	−0.0012 (7)	0.0023 (6)	0.0026 (7)
C37	0.0148 (8)	0.0162 (9)	0.0199 (8)	−0.0010 (7)	0.0028 (6)	0.0025 (7)
C38	0.0197 (8)	0.0196 (9)	0.0241 (9)	−0.0030 (8)	0.0035 (7)	−0.0018 (8)
O1S	0.0334 (7)	0.0312 (8)	0.0342 (7)	−0.0064 (7)	0.0098 (6)	−0.0028 (7)
C1S	0.0314 (11)	0.0397 (14)	0.0549 (14)	−0.0035 (11)	0.0113 (10)	−0.0164 (12)
O2S	0.0231 (6)	0.0184 (7)	0.0285 (7)	−0.0018 (6)	−0.0015 (5)	0.0021 (6)
C2S	0.0395 (13)	0.0212 (11)	0.087 (2)	−0.0006 (11)	−0.0315 (13)	0.0035 (13)
O3S	0.0184 (6)	0.0216 (7)	0.0205 (6)	−0.0016 (6)	0.0034 (5)	−0.0008 (6)
C3S	0.0197 (8)	0.0247 (10)	0.0280 (9)	0.0043 (8)	0.0034 (7)	0.0005 (9)
O4S	0.0155 (6)	0.0302 (8)	0.0243 (6)	−0.0002 (6)	0.0016 (5)	0.0002 (6)
C4S	0.0249 (9)	0.0278 (11)	0.0277 (9)	0.0022 (9)	0.0056 (7)	0.0017 (9)

### Geometric parameters (Å, º)

**Table:**

O1—C19	1.202 (2)	C13—C14	1.533 (2)
O2—C19	1.351 (2)	C14—H14A	0.9900
O2—C21	1.427 (2)	C14—H14B	0.9900
O3—C22	1.415 (2)	C15—C16	1.511 (3)
O3—H3O	0.8400	C15—H15A	0.9900
O4—C23	1.428 (2)	C15—H15B	0.9900
O4—H4O	0.8605	C16—C17	1.328 (3)
O5—C24	1.419 (2)	C17—H17A	0.9500
O5—H5O	0.8400	C17—H17B	0.9500
O6—C21	1.411 (2)	C18—H18A	0.9800
O6—C25	1.441 (2)	C18—H18B	0.9800
O7—C26	1.423 (2)	C18—H18C	0.9800
O7—H7O	0.8400	C20—H20A	0.9800
O8—C27	1.401 (2)	C20—H20B	0.9800
O8—C13	1.443 (2)	C20—H20C	0.9800
O9—C33	1.393 (2)	C21—C22	1.514 (2)
O9—C28	1.429 (2)	C21—H21	1.0000
O10—C29	1.4272 (19)	C22—C23	1.531 (2)
O10—H10O	0.8400	C22—H22	1.0000
O11—C30	1.435 (2)	C23—C24	1.525 (2)
O11—H11O	0.8400	C23—H23	1.0000
O12—C27	1.417 (2)	C24—C25	1.529 (2)
O12—C31	1.4360 (19)	C24—H24	1.0000
O13—C32	1.429 (2)	C25—C26	1.513 (2)
O13—H13O	0.8400	C25—H25	1.0000
O14—C34	1.415 (2)	C26—H26A	0.9900
O14—H14O	0.8400	C26—H26B	0.9900
O15—C35	1.428 (2)	C27—C28	1.531 (2)
O15—H15O	0.8400	C27—H27	1.0000
O16—C36	1.431 (2)	C28—C29	1.527 (2)
O16—H16O	0.8400	C28—H28	1.0000
O17—C33	1.425 (2)	C29—C30	1.524 (2)
O17—C37	1.433 (2)	C29—H29	1.0000
O18—C38	1.417 (2)	C30—C31	1.532 (2)
O18—H18O	0.8400	C30—H30	1.0000
C1—C2	1.530 (2)	C31—C32	1.513 (2)
C1—C10	1.548 (2)	C31—H31	1.0000
C1—H1A	0.9900	C32—H32A	0.9900
C1—H1B	0.9900	C32—H32B	0.9900
C2—C3	1.520 (3)	C33—C34	1.521 (2)
C2—H2A	0.9900	C33—H33	1.0000
C2—H2B	0.9900	C34—C35	1.523 (2)
C3—C4	1.545 (2)	C34—H34	1.0000
C3—H3A	0.9900	C35—C36	1.524 (2)
C3—H3B	0.9900	C35—H35	1.0000
C4—C19	1.532 (2)	C36—C37	1.531 (2)
C4—C18	1.541 (3)	C36—H36	1.0000
C4—C5	1.566 (2)	C37—C38	1.515 (3)
C5—C6	1.534 (2)	C37—H37	1.0000
C5—C10	1.559 (2)	C38—H38A	0.9900
C5—H5A	1.0000	C38—H38B	0.9900
C6—C7	1.530 (2)	O1S—C1S	1.400 (3)
C6—H6A	0.9900	O1S—H1OS	0.8400
C6—H6B	0.9900	C1S—H1S1	0.9800
C7—C8	1.533 (2)	C1S—H1S2	0.9800
C7—H7A	0.9900	C1S—H1S3	0.9800
C7—H7B	0.9900	O2S—C2S	1.414 (3)
C8—C14	1.551 (2)	O2S—H2OS	0.8400
C8—C9	1.553 (2)	C2S—H2S1	0.9800
C8—C15	1.558 (2)	C2S—H2S2	0.9800
C9—C11	1.552 (3)	C2S—H2S3	0.9800
C9—C10	1.573 (2)	O3S—C3S	1.445 (2)
C9—H9	1.0000	O3S—H3OS	0.8400
C10—C20	1.541 (2)	C3S—H3S1	0.9800
C11—C12	1.543 (2)	C3S—H3S2	0.9800
C11—H11A	0.9900	C3S—H3S3	0.9800
C11—H11B	0.9900	O4S—C4S	1.429 (2)
C12—C13	1.530 (2)	O4S—H4OS	0.8400
C12—H12A	0.9900	C4S—H4S1	0.9800
C12—H12B	0.9900	C4S—H4S2	0.9800
C13—C16	1.522 (2)	C4S—H4S3	0.9800
			
C19—O2—C21	116.36 (14)	O2—C21—H21	110.3
C22—O3—H3O	109.5	C22—C21—H21	110.3
C23—O4—H4O	121.8	O3—C22—C21	106.71 (14)
C24—O5—H5O	109.5	O3—C22—C23	112.11 (15)
C21—O6—C25	111.74 (13)	C21—C22—C23	107.43 (13)
C26—O7—H7O	109.5	O3—C22—H22	110.2
C27—O8—C13	118.89 (13)	C21—C22—H22	110.2
C33—O9—C28	114.62 (13)	C23—C22—H22	110.2
C29—O10—H10O	109.5	O4—C23—C24	111.11 (14)
C30—O11—H11O	109.5	O4—C23—C22	111.60 (13)
C27—O12—C31	111.60 (13)	C24—C23—C22	110.75 (14)
C32—O13—H13O	109.5	O4—C23—H23	107.7
C34—O14—H14O	109.5	C24—C23—H23	107.7
C35—O15—H15O	109.5	C22—C23—H23	107.7
C36—O16—H16O	109.5	O5—C24—C23	111.83 (14)
C33—O17—C37	112.25 (12)	O5—C24—C25	106.70 (13)
C38—O18—H18O	109.5	C23—C24—C25	109.58 (14)
C2—C1—C10	113.84 (14)	O5—C24—H24	109.6
C2—C1—H1A	108.8	C23—C24—H24	109.6
C10—C1—H1A	108.8	C25—C24—H24	109.6
C2—C1—H1B	108.8	O6—C25—C26	107.76 (14)
C10—C1—H1B	108.8	O6—C25—C24	109.17 (13)
H1A—C1—H1B	107.7	C26—C25—C24	113.97 (15)
C3—C2—C1	110.58 (16)	O6—C25—H25	108.6
C3—C2—H2A	109.5	C26—C25—H25	108.6
C1—C2—H2A	109.5	C24—C25—H25	108.6
C3—C2—H2B	109.5	O7—C26—C25	113.42 (14)
C1—C2—H2B	109.5	O7—C26—H26A	108.9
H2A—C2—H2B	108.1	C25—C26—H26A	108.9
C2—C3—C4	113.25 (15)	O7—C26—H26B	108.9
C2—C3—H3A	108.9	C25—C26—H26B	108.9
C4—C3—H3A	108.9	H26A—C26—H26B	107.7
C2—C3—H3B	108.9	O8—C27—O12	105.51 (13)
C4—C3—H3B	108.9	O8—C27—C28	109.39 (13)
H3A—C3—H3B	107.7	O12—C27—C28	108.80 (13)
C19—C4—C18	106.17 (15)	O8—C27—H27	111.0
C19—C4—C3	108.65 (15)	O12—C27—H27	111.0
C18—C4—C3	107.26 (14)	C28—C27—H27	111.0
C19—C4—C5	115.91 (14)	O9—C28—C29	109.91 (13)
C18—C4—C5	109.64 (15)	O9—C28—C27	106.83 (13)
C3—C4—C5	108.87 (14)	C29—C28—C27	111.73 (13)
C6—C5—C10	110.67 (13)	O9—C28—H28	109.4
C6—C5—C4	116.57 (14)	C29—C28—H28	109.4
C10—C5—C4	116.05 (15)	C27—C28—H28	109.4
C6—C5—H5A	103.9	O10—C29—C30	109.91 (13)
C10—C5—H5A	103.9	O10—C29—C28	108.48 (13)
C4—C5—H5A	103.9	C30—C29—C28	111.57 (13)
C7—C6—C5	109.08 (14)	O10—C29—H29	108.9
C7—C6—H6A	109.9	C30—C29—H29	108.9
C5—C6—H6A	109.9	C28—C29—H29	108.9
C7—C6—H6B	109.9	O11—C30—C29	107.58 (13)
C5—C6—H6B	109.9	O11—C30—C31	110.68 (14)
H6A—C6—H6B	108.3	C29—C30—C31	111.33 (13)
C6—C7—C8	114.15 (15)	O11—C30—H30	109.1
C6—C7—H7A	108.7	C29—C30—H30	109.1
C8—C7—H7A	108.7	C31—C30—H30	109.1
C6—C7—H7B	108.7	O12—C31—C32	106.02 (14)
C8—C7—H7B	108.7	O12—C31—C30	109.55 (13)
H7A—C7—H7B	107.6	C32—C31—C30	111.61 (14)
C7—C8—C14	114.08 (14)	O12—C31—H31	109.9
C7—C8—C9	111.02 (13)	C32—C31—H31	109.9
C14—C8—C9	112.80 (15)	C30—C31—H31	109.9
C7—C8—C15	110.06 (15)	O13—C32—C31	112.51 (14)
C14—C8—C15	99.22 (13)	O13—C32—H32A	109.1
C9—C8—C15	108.96 (13)	C31—C32—H32A	109.1
C11—C9—C8	110.90 (13)	O13—C32—H32B	109.1
C11—C9—C10	114.91 (15)	C31—C32—H32B	109.1
C8—C9—C10	116.87 (14)	H32A—C32—H32B	107.8
C11—C9—H9	104.1	O9—C33—O17	107.95 (12)
C8—C9—H9	104.1	O9—C33—C34	108.18 (14)
C10—C9—H9	104.1	O17—C33—C34	108.45 (13)
C20—C10—C1	108.14 (15)	O9—C33—H33	110.7
C20—C10—C5	113.13 (14)	O17—C33—H33	110.7
C1—C10—C5	108.87 (14)	C34—C33—H33	110.7
C20—C10—C9	113.54 (13)	O14—C34—C33	112.18 (13)
C1—C10—C9	106.46 (13)	O14—C34—C35	108.56 (13)
C5—C10—C9	106.43 (14)	C33—C34—C35	108.75 (15)
C12—C11—C9	116.11 (14)	O14—C34—H34	109.1
C12—C11—H11A	108.3	C33—C34—H34	109.1
C9—C11—H11A	108.3	C35—C34—H34	109.1
C12—C11—H11B	108.3	O15—C35—C34	111.25 (15)
C9—C11—H11B	108.3	O15—C35—C36	108.94 (13)
H11A—C11—H11B	107.4	C34—C35—C36	109.21 (14)
C13—C12—C11	110.56 (15)	O15—C35—H35	109.1
C13—C12—H12A	109.5	C34—C35—H35	109.1
C11—C12—H12A	109.5	C36—C35—H35	109.1
C13—C12—H12B	109.5	O16—C36—C35	110.68 (14)
C11—C12—H12B	109.5	O16—C36—C37	109.22 (14)
H12A—C12—H12B	108.1	C35—C36—C37	109.37 (13)
O8—C13—C16	115.42 (13)	O16—C36—H36	109.2
O8—C13—C12	104.55 (14)	C35—C36—H36	109.2
C16—C13—C12	109.75 (14)	C37—C36—H36	109.2
O8—C13—C14	115.29 (13)	O17—C37—C38	106.37 (14)
C16—C13—C14	103.64 (15)	O17—C37—C36	109.80 (14)
C12—C13—C14	108.04 (13)	C38—C37—C36	112.43 (14)
C13—C14—C8	101.28 (13)	O17—C37—H37	109.4
C13—C14—H14A	111.5	C38—C37—H37	109.4
C8—C14—H14A	111.5	C36—C37—H37	109.4
C13—C14—H14B	111.5	O18—C38—C37	112.25 (14)
C8—C14—H14B	111.5	O18—C38—H38A	109.2
H14A—C14—H14B	109.3	C37—C38—H38A	109.2
C16—C15—C8	106.09 (14)	O18—C38—H38B	109.2
C16—C15—H15A	110.5	C37—C38—H38B	109.2
C8—C15—H15A	110.5	H38A—C38—H38B	107.9
C16—C15—H15B	110.5	C1S—O1S—H1OS	109.5
C8—C15—H15B	110.5	O1S—C1S—H1S1	109.5
H15A—C15—H15B	108.7	O1S—C1S—H1S2	109.5
C17—C16—C15	127.79 (17)	H1S1—C1S—H1S2	109.5
C17—C16—C13	125.76 (18)	O1S—C1S—H1S3	109.5
C15—C16—C13	106.39 (14)	H1S1—C1S—H1S3	109.5
C16—C17—H17A	120.0	H1S2—C1S—H1S3	109.5
C16—C17—H17B	120.0	C2S—O2S—H2OS	109.5
H17A—C17—H17B	120.0	O2S—C2S—H2S1	109.5
C4—C18—H18A	109.5	O2S—C2S—H2S2	109.5
C4—C18—H18B	109.5	H2S1—C2S—H2S2	109.5
H18A—C18—H18B	109.5	O2S—C2S—H2S3	109.5
C4—C18—H18C	109.5	H2S1—C2S—H2S3	109.5
H18A—C18—H18C	109.5	H2S2—C2S—H2S3	109.5
H18B—C18—H18C	109.5	C3S—O3S—H3OS	109.5
O1—C19—O2	122.83 (16)	O3S—C3S—H3S1	109.5
O1—C19—C4	125.09 (16)	O3S—C3S—H3S2	109.5
O2—C19—C4	111.98 (15)	H3S1—C3S—H3S2	109.5
C10—C20—H20A	109.5	O3S—C3S—H3S3	109.5
C10—C20—H20B	109.5	H3S1—C3S—H3S3	109.5
H20A—C20—H20B	109.5	H3S2—C3S—H3S3	109.5
C10—C20—H20C	109.5	C4S—O4S—H4OS	109.5
H20A—C20—H20C	109.5	O4S—C4S—H4S1	109.5
H20B—C20—H20C	109.5	O4S—C4S—H4S2	109.5
O6—C21—O2	105.37 (14)	H4S1—C4S—H4S2	109.5
O6—C21—C22	112.14 (14)	O4S—C4S—H4S3	109.5
O2—C21—C22	108.39 (13)	H4S1—C4S—H4S3	109.5
O6—C21—H21	110.3	H4S2—C4S—H4S3	109.5

### Hydrogen-bond geometry (Å, º)

**Table:**

*D*—H···*A*	*D*—H	H···*A*	*D*···*A*	*D*—H···*A*
O4*S*—H4*OS*···O13^i^	0.84	1.90	2.7330 (17)	169
O3*S*—H3*OS*···O11^i^	0.84	1.88	2.7115 (19)	170
O2*S*—H2*OS*···O8^ii^	0.84	1.90	2.7381 (19)	177
O18—H18*O*···O2*S*^iii^	0.84	1.86	2.6857 (19)	167
O15—H15*O*···O10^iv^	0.84	2.22	3.0000 (19)	154
O14—H14*O*···O18^v^	0.84	1.91	2.6983 (19)	157
O13—H13*O*···O7^iii^	0.84	1.91	2.7080 (19)	158
O11—H11*O*···O3*S*^vi^	0.84	1.89	2.7248 (17)	169
O10—H10*O*···O16^iv^	0.84	1.88	2.7217 (18)	177
O7—H7*O*···O1*S*^v^	0.84	1.96	2.777 (2)	166
O1*S*—H1*OS*···O1	0.84	2.08	2.860 (2)	154
O16—H16*O*···O4*S*	0.84	1.83	2.6558 (17)	168
O5—H5*O*···O3*S*	0.84	1.99	2.8081 (17)	166
O4—H4*O*···O15	0.86	2.38	3.2012 (17)	160
O3—H3*O*···O15	0.84	1.94	2.7569 (17)	165

Symmetry codes: (i) *x*−1, *y*, *z*; (ii) *x*−1, *y*+1, *z*; (iii) *x*+1, *y*−1, *z*; (iv) −*x*+2, *y*+1/2, −*z*+1; (v) *x*, *y*+1, *z*; (vi) −*x*+2, *y*−1/2, −*z*+1.
